# Molecular Detection of Panton Valentine Leukocidin Toxin in Clinical Isolates of *Staphylococcus aureus* from Kiambu County, Kenya

**DOI:** 10.1155/2020/3106747

**Published:** 2020-08-27

**Authors:** Sani Iliya, Jonathan Mwangi, Ronald Maathai, Mary Muriuki, Christopher Wainaina

**Affiliations:** ^1^Department of Biological Sciences, School of Pure and Applied Sciences, Mount Kenya University, Thika, Kenya; ^2^School of Pharmacy and Health Sciences, United States International University-Africa, Nairobi, Kenya; ^3^Department of Biochemistry, Mount Kenya University, Thika, Kenya; ^4^School of Pure and Applied Sciences, Mount Kenya University, Thika, Kenya; ^5^Hain Life Science East Africa Ltd., Nairobi, Kenya

## Abstract

Panton–Valentine leukocidin gene is produced by *Staphylococcus aureus*, and methicillin-resistant *Staphylococcus aureus* isolates as a pore-forming toxin is largely responsible for skin and soft tissue illnesses. MRSA produces PVL toxins through *luk*S and *luk*F proteins causing tissue necrosis by damaging membrane of the defense cells. Presence of PVL toxin was tested from the 54 *S. aureus* clinical isolates obtained from Thika and Kiambu Level 5 Hospitals, in Kiambu County, Kenya, by Geno Type® MRSA assay (Hain Life Science, Nehren, Germany). DNA was isolated from freshly harvested bacterial cultures by spin column using Geno Type DNA isolation kit. The detection of PVL toxins was performed by amplification of genomic DNA and by reverse hybridization that identifies PVL genes using Geno Type MRSA kit. Out of 138 samples that were collected from patients in Kiambu County, 54 *S. aureus* isolates were obtained, of which 14 (25.9%; 95% CI = 11.9–38.9) samples had PVL toxins. The isolates that were obtained from the female patients had a higher PVL toxin prevalence of 35.7%, while the isolates collected from the male patients had a lower prevalence of 15.4% (*P* = 0.09). The pediatrics department had the highest PVL gene prevalence compared to outpatient department and surgical units (*P* = 0.08). However, the age groups of patients and the hospital attended by patients showed no significant difference in terms of PVL gene prevalence (*P* = 0.26). Therefore, the patients' gender and hospital units were not significantly associated with PVL gene prevalence (*P* = 0.08). This study shows that PVL positive isolates occur in the sampled hospitals in the county and female as well as children must be taken into consideration among patients with wound infections when isolating *S. aureus*.

## 1. Introduction

*Staphylococcus aureus* is a main human pathogen causing community acquired and hospital acquired infections resulting to widespread morbidity and mortality. *S. aureus* causes surface colonization of skin and mucus membrane of healthy as well as carrier individuals as the significant predisposing factor for infection [1, 2]. *S. aureus* is a substantial contributor to the overall burden on healthcare systems due to its high mortality rates of around 20–30% [3]. This burden is compounded by life-threatening complications, including infective endocarditis (IE) and other serious infections and complications that carry poor prognosis because of the anatomic site or the difficulty in reaching a timely diagnosis [4]. As part of its pathogenesis, *S. aureus* produces several virulence factors [5]. Panton–Valentine leukocidin (PVL) gene defined in 1932 by Panton and Valentine is a virulence factor produced by some strains of *S. aureus,* from two genes (lukS-PV and lukF-PV) encoding two proteins that causes leukocyte lysis and tissue necrosis [5–9]. PVL is associated with skin and soft tissue infection carried by both community-associated methicillin-susceptible *S. aureus* (CA-MSSA) and methicillin resistant *S. aureus* (CA-MRSA) and largely causes invasive as well as skin and soft tissue infections [10].

Panton-Valentine leukocidin is a pore-forming toxin largely responsible for skin and soft tissue illnesses [7]. There is significant variation in prevalence of PVL among *S. aureus* and methicillin-resistant *S. aureus* (MRSA) infections worldwide. Globally, prevalence of PVL toxin has been reported to increase across different countries such as Japan, USA, China, Germany, Egypt, Nigeria, and Kenya ranging from 12 to 90% [11–13]. Various studies have reported PVL prevalence with France at 5%, Bangladesh at 14.3%, Saudi Arabia at 8.1%, UK at 8.1%, Gambia at 72.9%, Nigeria at 10.7%, and Ghana at 75% [1, 12, 14]. Data on PVL prevalence and its pathogenesis in *S. aureus* and MRSA disease have not been sufficiently shown to have common occurrence in Kenya [15, 16].

PVL, as a pore-forming gene, is linked to *S. aureus* pathogenesis where it causes tissue necrosis and leukocyte damage [7]. There is substantial distinction in PVL prevalence among *S. aureus* infections worldwide. In a study carried out in Iran by Goudarzi et al. [17], PVL producing *S. aureus* had prevalence of 29.2%, where males had 58.6% and females had 41.4%. PVL producing *S. aureus* strains has been found as a carriage in healthy person and from infections with prevalence of 1.4% and 38.5%, respectively [8]. Molecular methods used in detecting PVL include a single polymerase chain reaction (PCR) to identify *luk*S-PV and *luk*F-PV toxins [8]. Methods used to type PVL included multilocus sequence typing (MLST) as well as staphylococcus cassette chromosome mec (SCCmec) [18]. Other methods include PCR-based hybridization assay that detects PVL and *γ*-hemolysin genes of *S. aureus* using oligonucleotide probe [19]. In addition, Lina et al. [20] developed rapid and simple PVL and *γ*-hemolysin genes detection procedures for *S. aureus* to ease the previous methods that were cumbersome including oligonucleotide probes [19, 21–23]. Goudarzi et al. [17] analyzed the presence of *luk*S-PV and *luk*F-PV genes encoding compounds of PVL using PCR technique according to the procedure and protocol describe by Lina et al. [20]. This study focused on identifying PVL genes from both outpatient and inpatient attending pediatrics, male and female surgical units of Thika and Kiambu Level 5 Hospitals in Kiambu County, Kenya.

## 2. Materials and Methods

### 2.1. Study Design

This was a cross-sectional descriptive laboratory-based study involving inpatients and outpatients in two health facilities in Kenya, namely, Thika and Kiambu Level 5 Hospitals. The study was conducted between December 2017 and September 2018.

### 2.2. Study Area

Kiambu County is located within greater Nairobi, with its capital being Kiambu town and its largest town is Thika. The county is very cosmopolitan and serves to provide residences for most of Nairobi's workforce. Thika Level 5 Hospital is a 300-bed Government Hospital in the town of Thika and Kiambu Level 5 Hospital is a 289-bed government hospital located in Kiambu, East District, Kiambu County [15].

### 2.3. Study Population

The population of Kiambu County is mixed urban and rural. The patients in this study were residents in the county, admitted or seeking services in the 2 health facilities.

### 2.4. Ethical Considerations and Approval

This study was given clearance by Ethical Review Committee of Mount Kenya University (No. MKU/ERC/0467). Approval was also obtained from National Commission for Science and Technology and Innovation (NACOSTI) and Nairobi Kenya (No. NACOSTI/P/17/23223/18191). In addition, approval was provided by the Ministry of Interior, County Commissioner, Kiambu County (No. ED.12/1/VOL.V/125), the Department of Health Services, County Government of Kiambu (No. KIAMBU/HRDU/AUTHO/2017/11/25/Iliya S), and Kiambu and Thika Level 5 Hospitals medical superintendents/Thika Level 5 Hospital Research Committee (No. MOMS/TKA VOL III (368)). All samples obtained were held and kept under maximum confidentiality with consent of the subjects or their guardians to participate in the study. All patients or their guardians who participated in this study signed or thumb-printed an informed consent form. This study was conducted in accordance with the Declaration of Helsinki.

### 2.5. Inclusion Criteria

Inclusion criteria included presentation with wound or purulent discharge during inpatient or outpatient services, children and adults irrespective of age, ability to understand risks and benefits of the study, consent and agreement by patient or guardians (in case of children), signing or thumb-printing informed consent form, and patients residing in the county and seeking services in the 2 health facilities.

### 2.6. Exclusion Criteria

Patients (or the guardians) who declined to sign the informed consent form and patients not residing in the county and not admitted to the two health facilities were excluded.

### 2.7. Sample Collection

Sterile dry sterile cotton swabs were used in the collection of samples from infected sites. After collection of samples, the swabs were placed in sheaths and transported to the laboratory for inoculation.

### 2.8. Culture of Samples

The samples were inoculated into 5% sheep blood agar plates obtained commercially from Kenya Medical Research Institute (KEMRI), Nairobi, and incubated at 37.0°C for 24 hours. Single colony of cultures from sheep blood agar were inoculated into mannitol salt agar (KEMRI, Nairobi) plates and incubated at 37.0°C for 24 hours before checking for fermentation of mannitol. All positive isolates from mannitol agar were subcultured into Mueller-Hinton agar (HiMedia, Mumbai, India) using a single discrete colony per plate (sample) and incubated at 37.0°C for 24 hours to obtain pure colonies. Identification of the bacterial isolates was carried out using standard microbiological procedures as was previously described including catalase test, coagulase, Gram staining, colony morphology, and fermentation of mannitol [24–26]. Pure cultures of *S. aureus* obtained were placed in tryptic soy broth (KEMRI, Nairobi, Kenya) and kept at −80°C until needed for further analysis. *S. aureus* ATCC-25923 was used as control.

### 2.9. Molecular Analysis

#### 2.9.1. Isolation and Purification of Genomic DNA from *S. aureus*

DNA was isolated from freshly harvested *S. aureus* cultures. Spin column DNA extraction procedure was used according to the technique developed by Hains Life Sciences using DNA isolation kit version 3.0® (Hain Lifescience, Nehren, Germany) for extraction of genomic DNA from *S. aureus* isolates. Five (5) colony forming units (CFU) of *S. aureus* suspended in 150 *μ*L of molecular biology grade water were used to harvest enough DNA for molecular analysis. The bacterial suspension was incubated at 95°C for 15 minutes in a heating block. The suspension was incubated again in ultrasonic bath for 15 minutes. The suspension was spin down in tabletop centrifuge at 1500 rpm for 5 minutes. To obtain supernatant solution of DNA, the spin filter membrane was washed with wash buffer to eliminate residual ethanol and DNA supernatant solution obtained after addition of elution buffer to the membrane. The DNA eluted was transferred to a new tube and stored at −20.0°C until needed for further analysis. Agarose gel electrophoresis was used to check for the DNA integrity of the DNA extracted.

#### 2.9.2. Procedure for Panton–Valentine Leukocidin (PVL) Toxins Detection

This was performed by amplification of genomic DNA followed by reverse hybridization that identifies PVL genes using Geno Type MRSA kit® (Hain Lifescience, Nehren, Germany) which was also designed to differentiate MRSA from *S. aureus* cultures. The kit contains a set of primers specific to staphylococcal cassette chromosome mec (SSCmec) types I, II, III, and IV including the community-acquired MRSA strain. The primers were designed to anneal to unique DNA regions and to generate amplicons that allowed identification of mecA and mecC that impart methicillin resistance as well as specific PVL gene fragments that give indication of CA-MRSA acquired in the environment. Amplification procedure was carried out in Thermocycler, ABI 2720 (Applied Biosystems, Weiterstadt, Germany), according to the Geno Type MRSA ver. 3 technique. PCR master mix was prepared in 45 *μ*L amounts and 5 *μ*L DNA solution added in a separate working area to make the final volume of 50 *μ*L. Detection of PVL was carried out by reverse hybridization using TwinCubator® (Hain Lifescience, Nehren, Germany) by means of a specific oligonucleotide probe targeting the SCCmec chromosomal cassette of MRSA that is immobilized on membrane strips. PCR amplicons hybridizes with probe during the detection process. Strips were added into individual troughs and hybridization carried out at 45°C for 30 minutes followed by two washings at 45°C for 30 minutes where amplicons that did not match were removed. Colorimetric identification of hybridized amplicons was performed by addition of streptavidin-conjugated alkaline phosphatase and the relevant substrate. The strips were air-dried and fixed on data sheet after final washings and read for the presence or absence of PVL genes. In comparison with standard/conventional method for PVL detection, this method achieved 100% correlation and shorter completion time [27, 28]. It is a rapid, sensitive, and specific method for the detection of PVL [28]. Standard hybridization with oligonucleotide probe method for detection PVL takes 14 hours as compared to GenoType based assay with 4 hours before PCR completion [19]. The procedure was controlled with reference DNA from *S. aureus* strain N315.

**Figure 1:**
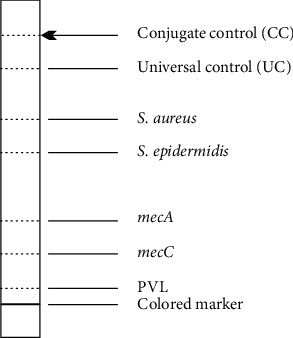
Geno Type MRSA ver. 3 target area Source: Hain Lifescience, Nehren, Germany.

Figure 1 shows the target area to be read on the strip.

### 2.10. Data Management and Statistical Analysis

Raw data was tabulated in Microsoft Excel, cleaned, and then exported to SPSS (Statistical Package for Social Science) software version 20.0 (IBM corporation, Armonk, New York, USA) for analysis. Values were expressed as percentages and frequencies. Categorical variables were analyzed using Chi square or Fisher exact tests (less than 5 expected counts in a cell) to test for the significant differences in their association. Differences were considered statistically significant when *P* < 0.05.

## 3. Results

### 3.1. Prevalence of *S. aureus*

Out of the 138 specimens obtained in the two hospitals in Kiambu County, Kenya, 54 samples were *S. aureus* culture positive giving a prevalence of 39.1% (95% CI = 30.1–46.4). Based on Hain Geno Type® assay, *mec*A impart methicillin resistance and the presence of bands on the target area confirmed the organism to be MRSA. Likewise, the presence of PVL genes gives indication of CA-MRSA strains that were acquired from the environment (Hain Lifescience leaflet and Geno Type manual). The presence of PVL in this assay, thus, serves to distinguish CA-MRSA from nosocomial MRSA. Among the 54 *S. aureus* isolates, 22 (40.7%) were found to be MRSA based on our earlier MRSA detection method by cefoxitin-based susceptibility [29].

### 3.2. Prevalence of Panton–Valentine Leukocidin Toxins

Presence of PVL genes was tested from the 54 *S. aureus* isolates by Geno Type® MRSA molecular assay. Fourteen of the 54 isolates tested positive for the PVL gene giving a prevalence of 25.9% (14/54; 95% CI = 17.5–39.1) as shown in Table 1. In addition, fourteen isolates out of the 54 *S. aureus* were *mec*A positive, one of which had dual PVL and *mec*A genes. More so, out of the 14 PVL positive *S. aureus* obtained, 9 were from the 22 MRSA (9/22) strains giving prevalence of 41% of PVL in MRSA strains.

#### 3.2.1. Prevalence of PVL Gene by Gender

There was no significant association between the gender of the patients and the PVL gene prevalence among *S. aureus* positive isolates in Kiambu County, Kenya (Table 1; Chi square = 2.901; d*f* = 1; *P*=0.09). The isolates that were obtained from the female patients had a PVL gene prevalence of 35.7% (10/28), while the isolates collected from the male patients had a prevalence of 15.4% (4/26) as shown in Table 1.

#### 3.2.2. PVL Gene Prevalence by Age Group in Years

**Figure 2:**
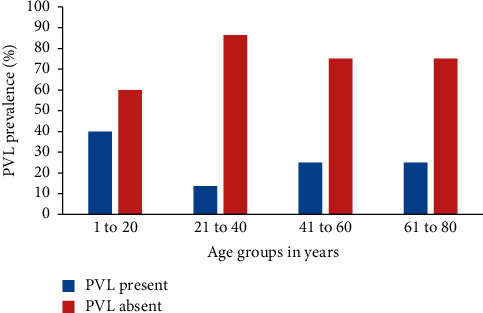
PVL gene prevalence by age groups in years.

There was no significant variation between the age groups of the patients in years and the prevalence of PVL gene in Kiambu County, Kenya (Table 1; Fisher's exact test; *P*=0.26). The age groups between 1 and 20, 21 to 40, 41 to 60, and 61 to 80 years old had a PVL gene prevalence of 40.0% (8/20), 13.6% (3/22), 25.0% (2/8), and 25.0% (1/4), respectively (Figure 2; Table 1).

#### 3.2.3. PVL Gene Prevalence by Hospitals

**Figure 3:**
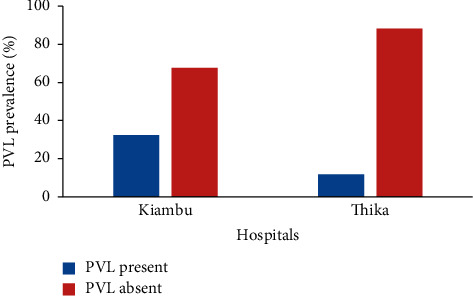
PVL gene prevalence by hospital.

There was no association between hospitals and PVL gene prevalence in Kiambu County, Kenya (Table 1; Fisher's exact test; *P*=0.18). Kiambu hospital had a PVL gene prevalence of 32.4% (12/37), while Thika hospital recorded a PVL gene prevalence of 11.8% (2/17) as shown in Figure 3 and Table 1.

#### 3.2.4. PVL Gene Prevalence by Hospital Units

**Figure 4:**
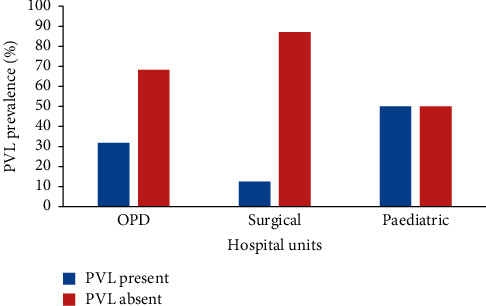
PVL gene prevalence by hospital units.

There was no significant association between hospital units and prevalence of PVL gene in Kiambu County, Kenya (Table 1; Fisher' exact test; *P*=0.08). The outpatient department (OPD), surgical and pediatric units, had a PVL gene prevalence of 31.8% (7/22), 12.4% (3/24), and 50% (4/8) as presented in Figure 4 and Table 1.

## 4. Discussion

The study has analyzed samples isolated from patients with purulent wounds for *S. aureus* in two very busy hospitals in Kiambu County, Kenya. The *S. aureus* positive samples were examined phenotypically through their growth in various media, Gram staining and confirmed by fermentation of mannitol, catalase, and coagulase tests. Out of 138 samples collected, 54 (39.1%) were observed to have *S. aureus*. Thus, the number of positive *S. aureus* obtained is more than that of study done in 2014 from inpatients by Aiken et al. [15] at Thika Level 5 Hospital (which is one of this study's sites) with a prevalence of 8.9% and 2013 by Mbogolori et al. [30], in the neighboring Nairobi County, who observed a prevalence of 20%.

Panton–Valentine leukocidin (PVL) toxin is linked to *S. aureus* infections where it causes tissue necrosis and leukocyte damage [7]. PVL is a pore-forming toxin largely responsible for skin and soft tissue illnesses [7]. In this study, we have tested the prevalence of PVL genes in 54 *S. aureus* positive clinical samples isolated from patients attending two major hospitals in Kiambu County, Kenya. Fourteen of the 54 isolates of *S. aureus* tested positive for the PVL gene which is a prevalence of 25.9%. This is higher than the prevalence observed in Spanish study between 2005 and 2008 with prevalence of 3% [31] and 4.5% in Malaysia by Nastaly et al. [32], as well as 20% observed by Owrang et al. [33] in Iran and 24.1% prevalence reported in China by Wu et al. [34]. The PVL prevalence observed in this study is also comparatively higher than those reported in some other African countries with Thabit et al. [35] reporting 5.4% prevalence in Egypt and prevalence of 10.7% in Nigeria by Orji et al. [13]. In a previous study in Kenya, Mbogolori et al. [30] observed a prevalence of 20% in Nairobi, while Aiken et al. [15] reported a prevalence of 7% from MSSA in Thika. However, a higher prevalence of 40% was reported by Shittu et al. [36] in Nigeria.

The prevalence of MRSA that harbor PVL in this study is 41% which is higher than that observed in many countries such as 5.3% in Malaysia [37], 24% in Bosnia and Herzegovina [38], 9.4% in Zambia [39], 29% in Libya [40], and 9% in South Africa [40]. This prevalence is even higher than that reported in 2013 from the neighboring Nairobi region, although the earlier study analyzed isolates from patients with specific conditions [16]. However, the prevalence is lower than the 56.8% reported in Nepal [41], 57.1% in Gambia [1], and 73% in Uganda [40]. In our analysis, we have observed a significant association between MRSA and PVL with 9 out of the 14 (64%; 95% CI = 0.35–0.87) PVL positive isolates also being MRSA. This suggests a possible selective advantage for the *S. aureus* strains carrying genes that confer drug resistance and virulence. This would spell doom for the current efforts to control and manage infections with *S. aureus* because healthcare would be overwhelmed with more virulent but also more drug-resistant infections. A broader study is therefore needed to investigate this association between drug resistance and virulence in *S. aureus* to forestall a potential epidemic.

The association between the gender of the patients and the PVL gene prevalence showed no statistically significant relationship in this study (Fisher's exact test; *P*=0.09). The isolates that were obtained from female patients had PVL gene prevalence of 35.7% compared to those collected from the male patients with a prevalence of 15.4%. Shallcross et al. [2] reported an association between gender and PVL-positive *S. aureus* carriage except that in their study males had the higher prevalence. The higher carriage of PVL in female as compared to male in our study could be due to the low sample size analyzed which could be explored further with higher sample size or bigger study.

In a study conducted by Darboe et al. [1] in Eritrea, PVL prevalence was high across all age groups and, therefore, age was not considered as a good predictor of toxins occurrence. Similarly, in this study, the age groups of patients showed no significant association with carriage of *S. aureus* positive for PVL genes (*P* > 0.26).

There was no association between hospitals and PVL gene prevalence in our study region (Fisher's exact test; *P*=0.18). Also, there was no observed significant association between hospital departments and prevalence of PVL gene-positive *S. aureus* (Fisher' exact test; *P*=0.08). The pediatrics unit had the highest PVL gene prevalence of 50.0%, while surgical unit had the lowest PVL gene prevalence of 12.5%. The high prevalence of PVL in hospital units at both facilities could be further explored in bigger study with higher sample size.

## 5. Conclusion

Methicillin-resistant *Staphylococcus aureus* presently poses a significant threat to public health mediations in both hospital and community settings globally. The results of our study have suggested that the prevalence of PVL carriage in *S. aureus* might be increasing compared to the levels observed in earlier studies in Kiambu County and the neighbouring Nairobi County. *S. aureus* PVL toxins could spread rapidly within the community leading to outbreak of community acquired infections. A greater concern, however, is the observed high PVL prevalence in MRSA *S. aureus* isolates. This needs to be further investigated in a broader study not only analyzing a bigger sample size but also incorporating patients with different health conditions. This will allow the exploration of this potential association of these two bacterial traits and what it portends for the management and control of *S. aureus* infections going forward.

The observed association of PVL-*S. aureus* carriage with gender and hospital departments, while not statistically significant in our study, needs to be further explored since they could impact the way infection control measures are practiced in different intrahospital settings. The overall results obtained in this study however need to be treated with caution. This is because our sample size is small and the participants were drawn from the geographical area served by the two hospitals sampled. The results therefore call for a bigger study, not just by increasing the sample size but by including other counties in the country to ascertain the findings. This would help in properly identifying the host and facility determinants that might predispose certain people to carry this more virulent form of the bacteria. However, it is imperative for the county's and health facilities authorities to take appropriate measures that include health education, surveillance, and screen all *S. aureus* isolates for PVL toxins so as to curtail outbreak of community as well as nosocomial infections. Hospital infection control strategy also needs to be strengthened in the county in order to prevent the spread of resistance.

## Figures and Tables

**Table 1:** Prevalence of the PVL gene.

Variable	*N*	PVL present	PVL absent	Chi square	*P* value
*Gender*					
Male	26	15.4% (4)	84.6% (22)	2.901	0.09
Female	28	35.7% (10)	64.3% (18)		

*Age*					
1–20	20	40.0% (8)	60.0% (12)	—	0.26
21–40	22	13.6% (3)	86.4% (19)		
41–60	8	25.0% (2)	75.0% (6)		
61–80	4	25.0% (1)	75.0% (3)		

*Hospital*					
Kiambu	37	32.4% (12)	67.6% (25)	—	0.18
Thika	17	11.8% (2)	88.2% (14)		

*Hospital unit*					
OPD	22	31.8% (7)	68.2% (15)	—	0.08
Surgical	24	12.5% (3)	87.5% (21)		
Paediatric	8	50.0% (4)	50.0% (4)		

Total	**54**	**25.9% (14)**	**74.1% (40)**		

Value expressed as percentage and frequencies (values in brackets). Chi square or Fisher's exact test was used to compute for associations between different groups. The *P* values <0.05 were considered significant. OPD = outpatient department, *N* = total number of samples; PVL = Panton–Valentine leukocidin.

## Data Availability

The data used to support the findings of this study are available from the corresponding author upon request.

## Abbreviations

CA-MRSA: Community-acquired methicillin resistant *S. aureus*
CA-MSSA: Community-associated methicillin-susceptible *S. aureus*
CFU: Colony forming units
IE: Infective endocarditis
MLST: Multilocus sequence typing
MRSA: Methicillin-resistant *S. aureus*
PCR: Polymerase chain reaction
PVL: Panton–Valentine leukocidin
*S. aureus*: *Staphylococcus aureus*
SCCmec: *Staphylococcus* cassette chromosome mec.
